# The time course of Temporal Binding in social and nonsocial interactions

**DOI:** 10.3758/s13423-024-02540-1

**Published:** 2024-07-17

**Authors:** Crystal A. Silver, Benjamin W. Tatler, Ramakrishna Chakravarthi, Bert Timmermans

**Affiliations:** School of Psychology, William Guild Building, Kings College, Old Aberdeen, AB24 3FX UK

**Keywords:** Temporal binding, Social interaction, Social cognition, Sense of agency

## Abstract

Temporal Binding (TB) is the subjective compression of action-effect intervals. While the effects of nonsocial actions are highly predictable, it is not the case when interacting with conspecifics, who often act under their own volition, at a time of their choosing. Given the relative differences in action-effect predictability in non-social and social interactions, it is plausible that TB and its properties differ across these situations. To examine this, in two experiments, we compared the time course of TB in social and nonsocial interactions, systematically varying action-effect intervals (200–2,100 ms). Participants were told they were (a) interacting with another person via a live webcam, who was in fact a confederate (social condition), (b) interacting with pre-recorded videos (nonsocial condition), or (c) observing two pre-recorded videos (control condition; Experiment 2). Results across experiments showed greater TB for social compared to nonsocial conditions, and the difference was proportional to the action-effect intervals. Further, in Experiment 1, TB was consistently observed throughout the experiment for social interactions, whereas nonsocial TB decreased from the first to the second half of the experiment. In Experiment 2, the nonsocial condition did not differ from control, whereas the social condition did, exhibiting enhanced binding. We argue these results suggest that the sociality of an interaction modulates the ‘internal clock’ of time perception.

**Supplementary Information:**

The online version contains supplementary material available at 10.3758/s13423-024-02540-1.

## Introduction

Temporal Binding (TB) is the perceived compression of time between an action and its effect (Beck et al., 2017; Buehner, 2012a; Engbert et al., 2007). Measuring TB over different action-effect intervals allows us to map the time course of TB for any given interaction. Investigation into the time course of *nonsocial* TB has revealed divergent results, with some studies reporting the strongest binding at the shortest delays (Haggard et al., 2002; Ruess et al., 2018), and others reporting binding effects increasing with interval length (Buehner & Humphreys, 2009; Dewey & Knoblich, 2014; Ruess et al., 2017; Wen et al., 2015). The reason for this divergence is unknown, but could simply be due to methodological differences: the specific set of intervals used and how the intervals were measured (Silver et al., 2021). TB effects have been shown to diverge depending on if they are measured by the Libet Clock method (i.e., timepoint judgements for action and effect on an analogue clockface) or interval estimation (Siebertz & Jansen, 2022). Specific parameters of the measure used have been found to influence TB effects (Ivanof, Terhune, Coyle, Gottero, et al., 2022a, Ivanof, Terhune, Coyle, Moore et al., 2022b). Further, individual components of TB, namely action and outcome binding (Hon, 2023), may be uncorrelated (Siebertz & Jansen, 2022; Tonn et al., 2021) with different underlying mechanisms (Tanaka et al., 2019).

More importantly, contradictions may arise because TB has contributory factors, which have not been scrutinised, nor accounted for, in the studies. For example, Humphreys and Buehner (2009) demonstrate a perceived shortening of time even between two non-causally related events. Whilst acknowledging these limitations, we still used TB as the measure throughout this study as we focused on differences in TB between two conditions where methodology outside of the experimental manipulation was consistent, and therefore any other influences would affect both conditions equally. We do not hypothesise about the exact mechanisms through which social and nonsocial contexts affect TB and acknowledge that a change in social context could affect Sense of Agency (SoA) and/or other processes involved in event timing (e.g., causation, Buehner, 2012b; attention, Vogel et al., 2021; multisensory integration, Kirsch et al., 2019; intention, Lorimer et al., 2020). Indeed, there is ongoing debate about how causation and intentionality (of actions) influence TB. Lorimer et al. (2020) argue that whilst causation underlies TB effects, the magnitude of those effects is modulated by intentionality.

The time course of social TB remains, to our knowledge, unresearched. This is unfortunate as social interactions are qualitatively different from nonsocial ones because of agency in the other (Pfeiffer et al., 2014; Stephenson et al., 2018). This is because in most nonsocial circumstances, cause and effect are temporally and experientially/observably contiguous (Cravo et al., 2011), whereas in social interactions this is not so. For example, if you flip a light switch, you expect the bulb to illuminate almost instantaneously. If you wave at another person, however, you understand that they wave back under their own volition, at a time of their choosing (if at all), so temporal expectations are not so clear cut.

Vogel et al. (2021) demonstrated that there is an enhancement of more TB in social, compared to nonsocial, contexts generally. This was true whether the social context was introduced by top-down (beliefs) or bottom-up information (stimuli). This social TB enhancement is supported by the wider literature, observed in a range of circumstances where participants interact with a human partner (Grynszpan et al., 2019; Obhi & Hall, 2011; Pfister et al., 2014) or on-screen face (Stephenson et al., 2018; Ulloa et al., 2019). Although the time course of social TB remains unknown, Pfeiffer et al. (2012) scrutinised the time course of social (explicit) SoA (i.e., Social Agency). While there is ongoing debate about what TB represents (see Buehner, 2012a, 2012b; Hoerl et al., 2020; Kirsch et al., 2019; Suzuki et al., 2019), the findings of Pfeiffer et al. (2012) shed light on the potential time course of social TB. They found that when the ‘other’ in an interaction always performed the same action, explicit SoA ratings diminished as action-effect intervals increased after peaking at 400 ms. It is reasonable to posit, therefore, that such social action-effect intervals will modulate the time course of TB, differently to the way nonsocial interactions do.

Foundational work in the field has established that (a) action-effect intervals modulate TB in nonsocial contexts, (b) there is a time course for *explicit* Social Agency, and (c) social contexts enhance TB compared to nonsocial contexts. It remains unknown whether, and how, the time course of social and nonsocial TB differs. Nevertheless, as reviewed above, the existing work permits some tentative predictions for the time course of social TB. Firstly, that there may be a peak of TB effects, like those found for an explicit measure of social SoA by Pfeiffer et al. (2012). Secondly, if there is a peak, it would be at a longer action-effect interval duration for the social, compared to nonsocial, condition. This is because almost immediate action-effect contiguity is expected in nonsocial circumstances (Cravo et al., 2011), whereas in social interaction this is not so (Pfeiffer et al., 2014; Stephenson et al., 2018). Thirdly, that the social condition would exhibit more TB compared to the nonsocial condition overall, as per the ‘social prioritisation’ effects described by Vogel et al. (2021). To this end, our study used a simple interaction paradigm to systematically manipulate both action-effect intervals and participants’ beliefs about interacting in social or non-social situations.

## General methods

We conducted two experiments. Experiment 1 was designed to investigate if whether a visual response (i.e., on-screen keypress) was socially derived or not would affect the time course of TB, and if this effect was contingent on a specific temporal contiguity of the response. Participants initiated an interaction at a time of their choice through a keypress, then watched a keypress by the ‘other’ after various intervals. The current study used videos of hands on-screen as stimuli so that the social condition could look like a live interaction (similar to Pfister et al.’s (2014) social TB study where there were two humans interacting), but with the advantage of maintaining experimental control. In the social condition, participants were told they were interacting directly with another person through keypresses streamed ‘live’ between webcams (each displayed on the other’s screen). In the nonsocial condition they were told that the reactions were pre-recorded videos; in reality, participants were always watching video recordings. After watching each video, participants were asked to replicate the interval between their keypress and the observed on-screen reaction (as in Vogel et al., 2021, 2022) by pressing and holding the spacebar for the action-reaction interval duration. Experiment 2 included a within-subjects control condition to account for individual differences in baseline interval replication. That is, participants completed both an active experimental condition (social or nonsocial) and a passive observational control condition. In the control condition participants watched two consecutive keypresses on-screen and replicated the interval between them, rather than initiating an action-response sequence.

The interval replication measurement method was chosen for three reasons. Firstly, replication is a more intuitive method compared to estimation and required minimal practice and no training block. This was crucial in managing the testing session length; increased session length would increase the chance of participants becoming dubious of the ‘live’ interaction in the social condition. Secondly, pressing and holding the spacebar to replicate intervals allowed for scrutiny of very short interval durations, which would have been difficult to reproduce using the replication method where keypresses mark the start and end of the interval. Finally, Libet clock measures were not feasible for this design when they required visual attention, which would have competed with the on-screen stimulus.

Taken together, these experiments allowed us to directly compare the time course of social and nonsocial TB.

## Experiment 1

### Methods

#### Participants

Seventy-one students (52 females; age 18–52 years; mean age = 25.2 years; AQ scores 6–33, mean AQ = 17.1) from the University of Aberdeen participated for renumeration or course credit. Written informed consent was obtained from all participants. The study was approved by the Psychology Ethics Committee, University of Aberdeen (PEC/4344/2019/10). The only a priori inclusion criterion was that participants were naïve to the purpose of the study, i.e., they had not participated in previous similar studies from the research group. Based on medium effect size expectations in line with Vogel et al. (2021) Exp. 2 (*ƞ2* = .135 for their equivalent of Socialness), sample size was calculated with G*Power for a standard mixed-design 2 x 20 (Socialness x Interval) ANOVA (repeated measures: within-between interaction) for a medium effect size of Socialness (*f* = .25) and .8 power and a small interaction effect of Socialness*Interval (*f* = .10) and .8 power, recommending a total sample size of N = 68/64, respectively; hence we tested approximately 70 participants.

#### Design

The experiment used a 2 x 20 mixed-design with one between-subjects factor of ‘Socialness’ (‘live’ interaction = social condition, pre-recorded videos = nonsocial condition) and one randomised within-subjects factor of ‘Interval’ (200–2,100 ms in 100-ms increments; 20 conditions total). The Socialness factor was conducted as between-subjects for two reasons: to keep the duration of experimental sessions reasonable (< 1 h) and to maintain the necessary deception element: the same pre-recorded videos were used in both Socialness conditions, so participating in both could easily lead to the discovery that the social interactions were not ‘live’.

#### Materials and apparatus

The experiment took place in two testing cubicles in a research laboratory, each equipped with a desktop PC, LCD monitor, keyboard, and webcam. Each webcam was connected to the PC in the other cubicle so that it captured a video of the other cubicle’s keyboard and section of desk. It was positioned so that the interaction partner’s hand was exactly as seen in the experiment videos. E-Prime software (Schneider et al., 2002) was used for the delivery of the experimental protocol.

Colour videos of the interaction partner’s (i.e., confederate’s) button presses were pre-recorded using an iPhone 11’s default camera (Apple, 2021). Each video showed the partner’s hand with the index finger in a resting position on a keyboard, pressing the ‘B’ key and then returning to the original position. Videos were edited using VideoPad Video Editor for Mac (NCH Software, 2020) to be 11 s long. The hand is seen at rest until the keypress occurs at exactly 10 s, and the hand returns to resting position for the remaining second. Importantly, the hand was required to remain as still as possible before initiating the move to press the ‘B’ key. This allowed the E-Prime programme (Schneider et al., 2002) to cut sections of the video seamlessly (i.e., without the hand position jumping), as needed for the specific time intervals between participant and on-screen keypresses. Multiple videos were recorded with slightly different hand starting positions to increase believability in the ‘live’ condition that the videos were in fact a genuine live stream from the partner’s webcam.

Participants filled out the Autism Spectrum Quotient (AQ; Baron-Cohen et al., 2001) questionnaire. This 50-item self-report questionnaire evaluates the degree to which an individual possesses traits associated with Autism Spectrum Disorder, with higher scores (range 0–50) indicating the presence of more autistic traits; any score of 32+ is of clinical relevance (Baron-Cohen et al., 2001). It has been demonstrated that higher AQ scores can be indicative of lower sensitivity to social stimuli (Bayliss et al., 2005; Freeth et al., 2013). The sample did not include anyone in the clinical range (mean = 17.19, SD = 6.42).

#### Procedure

**Fig. 1:**
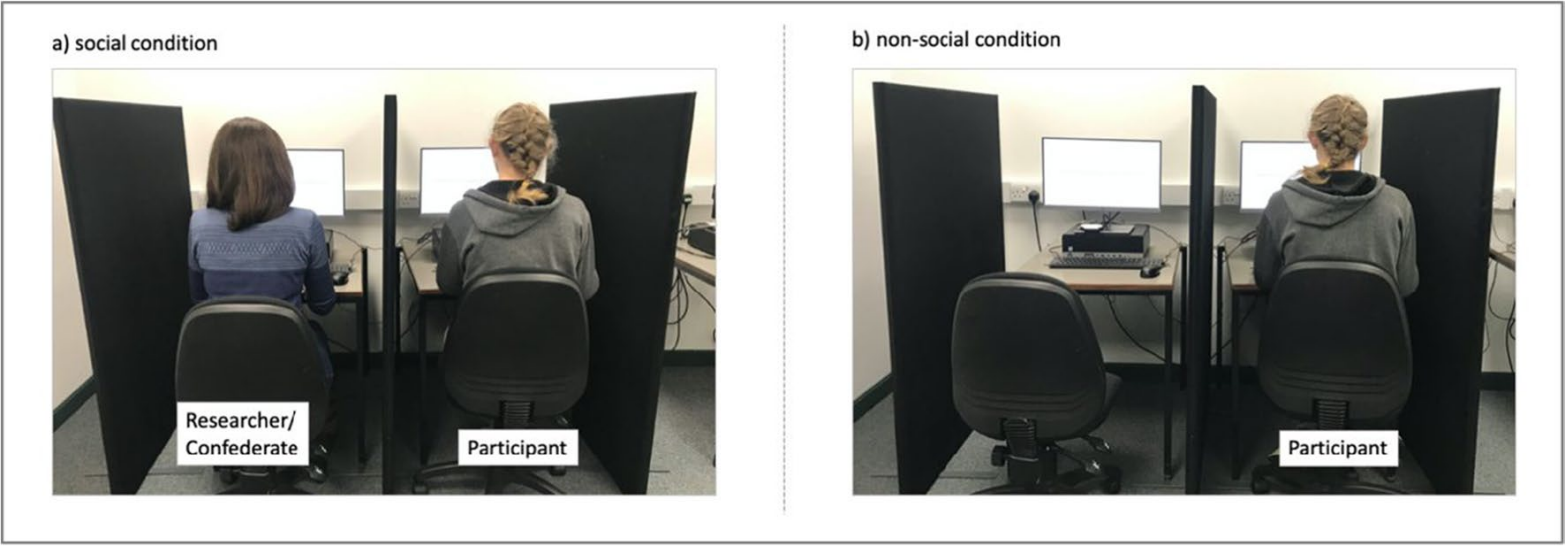
Experimental setup in Experiment 1: (**a**) an image of the social condition where the confederate (i.e., researcher) was seated at the adjacent testing cubicle, and (**b**) an image of the nonsocial condition where the participant was seated alone

Participants were randomly assigned to either the ‘live’ social condition or the nonsocial condition. In the social condition, participants were told that they would be interacting with the researcher who would respond to them at a time of their choosing, whereas in the nonsocial condition they were told that they would be watching pre-recorded videos that would be played in response to their action. Hence, the experimental task was completed by the participant as a pair (i.e., the participant and the researcher) in the social condition, or individually in the nonsocial condition (see Fig. 1).

To improve believability of the deceptive interaction, in the social condition the researcher pre-launched the webcam feed before participants entered the laboratory. Then, the researcher explained the webcam set-up for viewing each other’s hands and demonstrated this by waving their hand in front of one camera, letting the participant view this action on the other cubicle’s PC monitor. Importantly, this genuinely live feed was left open in a background window throughout the experiment and the researcher kept their hand in place at the ‘B’ key of the keyboard. This also enhanced believability because when E-Prime automatically terminated, the webcam would once again be in view, with the hand in a convincingly similar position to what had just been viewed in trials. In the nonsocial condition, the researcher was present in the lab throughout the testing session, but worked on other tasks at a workstation in the opposite corner of the room and only interacted with the participant during instruction and debrief (unless the participant had any questions, which were answered as needed). All participants were instructed that the purpose of the experiment was to see how accurate they could be when replicating the time interval between their action and the viewed response; accuracy was emphasised. Participants in the social condition were instructed not to move the position of the keyboard and to keep their hand as still as possible when not pressing the ‘B’ key. This was explained as being important to reduce ambiguity about when they pressed the key or observing the other’s keypress, but was in fact a cover story to justify why the hand viewed on-screen was (perhaps unusually) still.

**Fig. 2:**
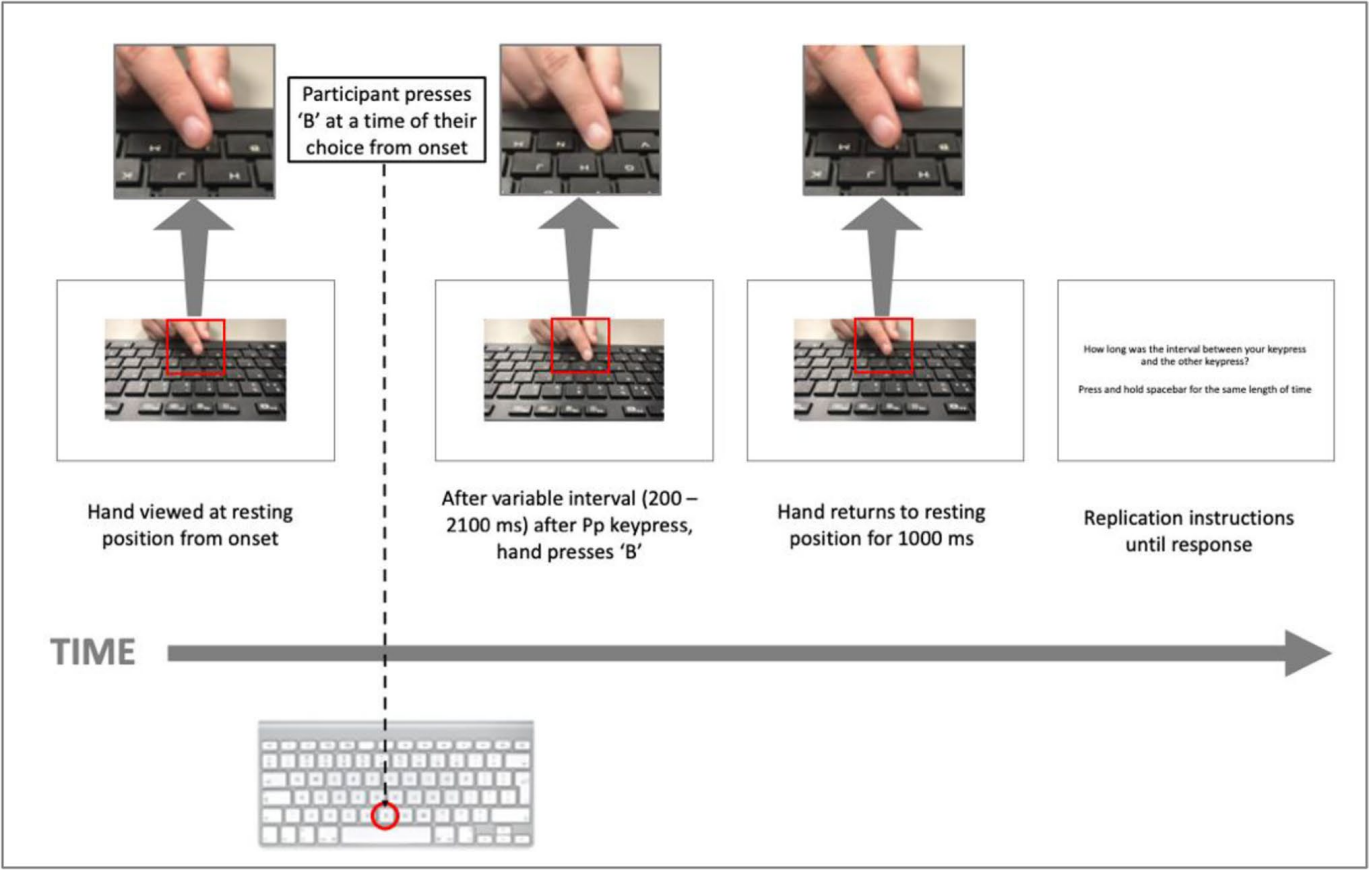
A schematic representation of the trial sequence: Trial begins with the participant viewing the resting hand of the ‘other’ on-screen; the participant then presses ‘B’ on the keyboard at a time of their choice; the on-screen hand then presses ‘B’ after a variable interval and returns to resting position; the interval replication instructions are displayed. Magnified inset areas from the screenshots show details of the other’s button press

On each trial, the participant pressed ‘B’ on their keyboard at a time of their choice. The (partner’s) hand on-screen would press ‘B’ in response after a variable time interval (200–2,100 ms in 100-ms increments; randomised across trials). The on-screen hand was visible from trial onset to 1,000 ms after the hand pressed ‘B’. Following each keyboard interaction, the participant was asked to replicate the interval between their keypress and the on-screen keypress by pressing and holding the spacebar for the same length of time (see Fig. 2 for trial sequence). After a 500-ms blank screen, the next trial began.

The experiment started with a practice block of ten trials where only the participant’s action and the on-screen response were presented. The participant was not required to do any task. In a further 20 trials this sequence was followed by the replication task, where the participant replicated the perceived interval between their keypress and the response keypress. Two experimental blocks (40 trials; each ‘Interval’ presented twice per block; 80 total) with the full sequence were then presented. Each block was self-paced allowing for breaks if desired. There was never any feedback given to participants about the accuracy of their replications throughout the entire experiment (as in Stephenson et al., 2018).

After completing the experimental task, participants completed the AQ (Baron-Cohen et al., 2001). Participants in the social condition were asked, post-debrief, to report the strength of their belief that they were interacting with the other person using a 5-point Likert scale (extremely/very/somewhat/slightly/not at all).

#### Statistical analysis

The data were analysed with a mixed linear model (MLM; Kliegl et al., 2012).

##### Outlier detection and exclusion criteria for participant-wise and trial-wise measures

First, we excluded participants for whom the correlation coefficient between the presented intervals and the corresponding interval replications was more than 2.5 absolute deviations from the group median correlation coefficient (MAD; *b* = 1.4826; Leys et al., 2013) (two participants excluded). This ensured that participants included in the analysis had engaged with the task. Second, individual trials where responses were more than 2.5 absolute deviations from each *interval group median response* were excluded (MAD; *b* = 1.4826; Leys et al., 2013) (8.4% of trials were excluded). This removed response outliers. Finally, if any participant had more than 25% of their trials removed by the trial-wise criterion, they were excluded entirely from analyses (a further five participants excluded). Participants included in analysis after all exclusions were: 36 in the social condition (30 females; age 18–30 years; mean age = 22.2 years; AQ scores 6–33, mean AQ = 17.1), and 28 in the nonsocial condition (19 females; age 18–52 years; mean age = 25.2 years; AQ scores 8–33; mean AQ = 17.2).

##### Percentage temporal binding measure

All analyses were done on *percentage* TB, calculated as per Eq. 1 below. As TB is the perceived compression of time between events, a binding score below zero represents under-replication of an interval, zero represents accurate interval replication, and a score above zero represents an over-replication of an interval. Using percentage binding, rather than a measure of TB in milliseconds (i.e., $$interva{l}_{estimate}-interva{l}_{actual})$$, allows comparison across all intervals, in that it controls for the part of the Socialness x Interval effect that is due to increasing intervals trivially yielding larger replication errors. Importantly, whilst percentage TB was used for the above reason, interval replications were never related to divergence from ‘actual’ durations. Analyses focused on condition differences, i.e., temporal magnitude differences between experimental manipulations (as in Pfister et al., 2014; Vogel et al., 2021).

1 $${Binding}_{percentage}= \frac{interva{l}_{estimate}- interva{l}_{actual}}{interva{l}_{actual}} \times 100$$

##### Introducing belief, block and methodology as exploratory fixed effects

There were two fixed effects associated with experiment design (2 Socialness conditions x 20 Intervals) in the MLM. We included three additional factors in the model. First, we separated participants in the social condition into those who said they believed they were interacting with another person (N = 22) and those who said they didn’t (N = 14). This grouping was based on the responses to the ‘belief’ question at the end of the experiment. This resulted in the two-level between-subjects factor Socialness being replaced by a between-subjects *Socialness/Belief* factor with three levels (nonsocial/social_no/social_yes). Second, to explore the differences in the time course of TB across the experimental blocks, we introduced a factor *Block* with two levels (block 1/block 2). Third, to investigate if a change in the researcher who interacted with participants part way through testing influenced TB, we introduced a factor *Methodology* with two levels (1/2).^1^

##### MLM fixed and random effects structure

The fixed effects included in the MLM were as follows. A categorical fixed effect of ‘*Socialness/Belief’*. Contrasts were coded for this factor to compare nonsocial to all social participants (regardless of belief; *Socialness*) and the social believers to social non-believers (*Belief*). An ordered categorical fixed effect of Block (‘*BlockNr*’; 1,2) coded using orthogonal polynomial contrast. A continuous fixed effect of Interval (200–2,100 ms), which was centred and scaled using the base R *scale* function (‘*Interval_Scaled’;* R Core Team, 2019). A categorical fixed effect of ‘*Methodology*’. Contrasts were coded for this factor to compare conditions 1 and 2, which accounted for each researcher interacting with participants.^1^ The MLM was built using the *lmer* function of the *lme4* package (Bates et al., 2015) of *R* statistical programming language (R Core Team, 2019), in the *R Studio* integrated development environment (RStudio Team, 2019); ggplot2 (Wickham, 2016) was used for plotting data.

The strategy adopted when building the MLM was to begin with the maximal random effect structure justified by the design (i.e., include all random slopes and intercepts for within-subjects fixed effects, with interactions), then simplify the random effect structure element-by-element until the model converged (Barr et al., 2013). *‘Participant’* was included in the MLM as random effect; as ‘interval’ was a within-subject factor, it was important to include Participant to account for any individual differences within groups. This maximal model did not converge; we systematically simplified the random effect structure.

The random effect structure simplification strategy was to (1) drop interactions in slopes of random effects that accounted for the least amount of variance, (2) drop slopes of random effects, again starting with the slopes that accounted for the least amount of variance, and then, if necessary, (3) drop the intercept of the random effect that accounted for the least amount of variance (Barr et al., 2013). Once the maximal random effect structure that would converge was found, random effect structure simplification continued as above until the *optimal* random effect structure was reached. The optimal random effect structure was determined by comparing model versions through analysis of variance (ANOVA). Once a model had a significantly better fit than the next simpler model (i.e., the model with the next least contributing element removed), random effect structure simplification stopped. *lmer* (Bates et al., 2015) syntax of the optimal model is shown below:

$$Optimal\_model=lmer\;(Percent\_Binding\sim1+Interval\_Scaled\times Socialness/Belief\times BlockNr\times\text{Methodology}+\left(1+Interval\_Scaled\times BlockNr\left|Participant\right.\right),\;data=Exp12,\;control=lmerControl\;(optimizer=''bobyqa''))$$

The *p-*values for fixed effects within the MLM were generated using the summary() function of base R (R Core Team, 2019). This function has a default method of generating *p-*values through t-tests using Satterthwaite’s method ['lmerModLmerTest']; this was used throughout.

### Results

**Fig. 3:**
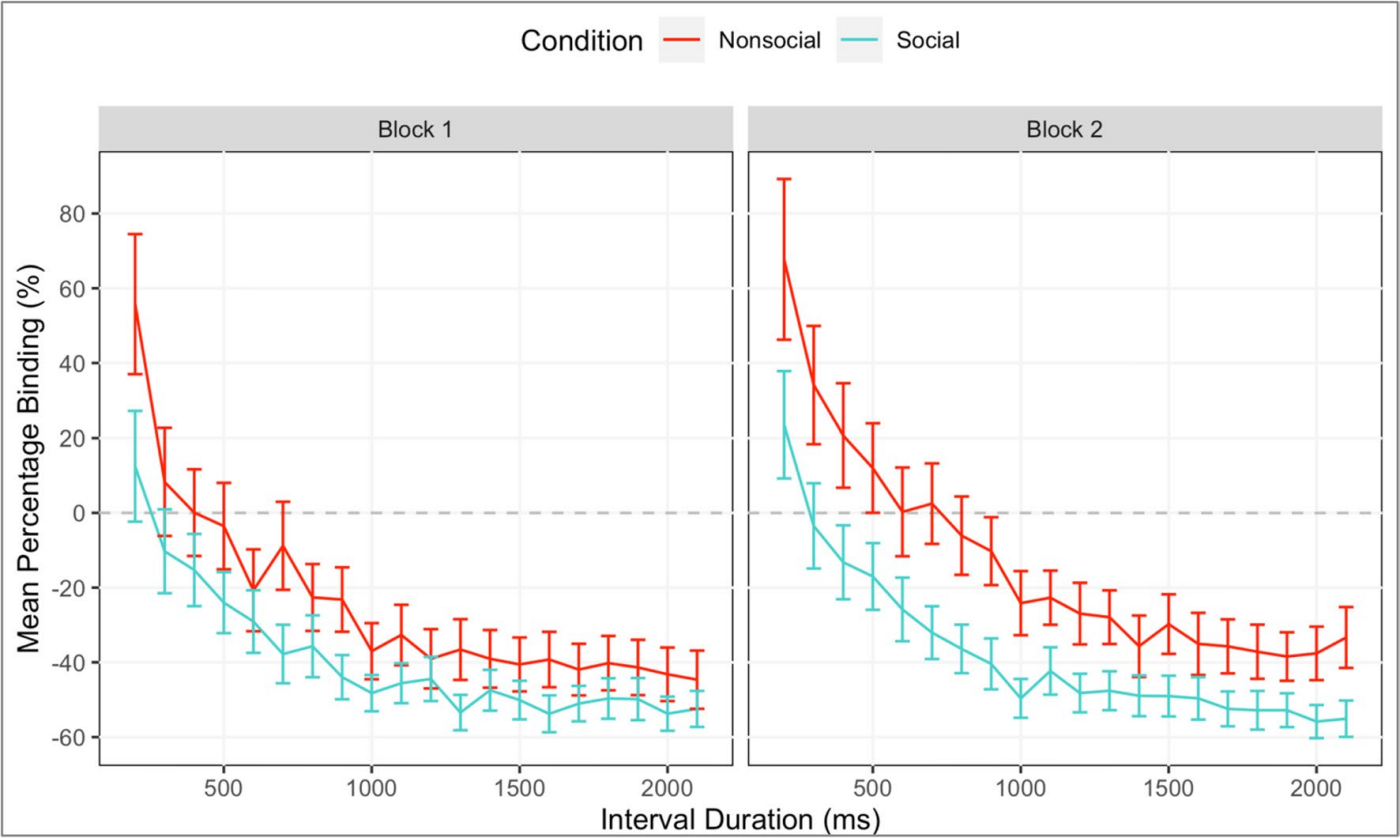
Temporal binding (TB; %) plotted as a function of interval. Mean TB % for social (green) and nonsocial (red) conditions are plotted separately within each block panel to highlight the significant socialness*block interaction in the mixed linear model. Error bars represent 95% confidence intervals

The output of the MLM is shown in Table 1. Crucially, there was a main effect of Socialness, with more percentage-binding in the social condition (*M = -38.4, 95% CI [-39.7,-37.1]*), compared to the nonsocial condition (*M = -19.4, 95% CI [-21.3,-17.5]*), *β= 20.52, SE = 4.54, t = 4.52, p < .001* (to rule out any contributory effects of differing participant demographics to this between-subject result, exploratory analyses were carried out regarding Age of participants: no significant results were found; see Online Supplementary Materials (OSM)). The effect of Socialness was qualified by an interaction with Interval (*β = -5.87, SE = 2.58, p = .027*), which we explored through curve fitting (see OSM), and with Block (*β = 6.87, SE = 1.98, t = 3.47, p = .001)*, with the effect of Socialness being larger in block 2 (see Fig. 3). There was no difference in binding between participants who said they believed the social interaction (*M = -38.4, 95% CI[-40.0,-36.8]*) and those who did not (*M = -38.5,95% CI[-40.7,-36.2]*), *β= .10,SE = 6.06,t = .002, p = .987*.

**Table 1:** Results of the mixed linear model to predict temporal binding for Experiment 1

**Fixed effect**		***β***		***SE***		***df***		***T***		***p***
Intercept		-31.63		2.31		57.90		-13.67		<.001
Methodology		-3.76		4.63		57.90		-0.81		0.421
Interval		-18.22		1.32		57.95		-13.85		<.001
Socialness		20.52		4.54		57.95		4.52		<.001
Belief		0.10		6.06		57.86		0.02		.987
Block		3.85		1.01		57.34		3.82		<.001
Interval*Methodology		-0.58		2.63		57.95		-0.22		.828
Interval*Socialness		-5.87		2.58		58.12		-2.27		.027
Interval*Belief		-3.68		3.44		57.82		-1.07		.289
Interval*Block		-2.42		0.61		4807.69		-3.99		<.001
Socialness*Block		6.87		1.98		57.85		3.47		.001
Belief*Block		2.40		2.64		56.96		0.91		.366
Methodology*Belief		-2.01		12.13		57.86		-0.17		.869
Methodology*Socialness		-0.72		9.09		57.95		-0.08		.937
Methodology*Block		0.94		2.02		57.34		0.46		.644
Interval *Socialness*Block		-0.77		1.20		4811.89		-0.64		.520
Interval*Belief*Block		0.85		1.58		4804.40		0.54		.590
Interval*Block*Methodology		-3.63		1.21		4807.69		-3.00		.003
Interval*Socialness* Methodology		0.04		5.17		58.12		0.01		.994
Interval*Belief*Methodology		-0.44		6.89		57.82		-0.06		.949
Block*Socialness*Methodology		-4.12		3.97		57.85		-1.04		.303
Block*Belief*Methodology		0.06		5.27		56.96		0.01		.990
Interval*Block*Socialness* Methodology		4.86		2.39		4811.89		2.03		.042
Interval*Block*Belief* Methodology		-4.43		3.16		4804.40		-1.40		.161
**Random effect**	**Element**		**Variance**		**SD**		**Corr.**			
Participant	Intercept		303.32		17.42					
	Interval(slope)		90.64		9.52		-0.25			
	Block(slope)		39.45		6.28		0.21		0.36	
	Interval*Block(slope)		10.48		3.24		-0.26		-0.20 -0.99

Percentage TB increased with Interval, *β= -18.22, SE = 1.32, t = -13.85, p < .001.* There was a significant main effect of Block on binding, *β= 3.85, SE = 1.01, t = 3.82, p < .001*, with responses in block 2 (*M = -27.4, 95% CI[-29.1,25.7]*) exhibiting less binding overall than block 1 (*M = -33.1, 95% CI[-34.6,-31.5]*).

To investigate the interaction between *Socialness and Block*, additional analyses were carried out. Follow-up MLMs were run to test the simple effects of Socialness (social or nonsocial) and Block. Within block 1, binding was significantly greater for the social, compared to nonsocial condition, *β= -15.10, SE = 4.42, t = -3.42, p = .001 (M = -39.3, 95% CI[-41.1,-37.5] and M = -24.8, 95% CI[-27.4,-22.3], respectively),* this was further enhanced in block 2, *β= -25.07, SE = 4.42,t = -5.67,p < .001 (M = -37.5, 95% CI[-39.4,-35.6] and M = -13.8, 95% CI[-16.5,-11.0], respectively).* As shown in Fig. 3, this increasing difference between social and nonsocial was due to a decrease in binding in the nonsocial condition from block 1 to block 2, *β= 11.68, SE = 1.27, t = 9.21, p < .001,* while there was no difference in the social condition from block 1 to block 2, *β= 1.71, SE = 1.10,t = 1.55,p = .121*.

### Discussion

Experiment 1 demonstrated enhanced TB effects in the social compared to the nonsocial condition, in line with previous studies (Grynszpan et al., 2019; Obhi & Hall, 2011; Pfister et al., 2014; Stephenson et al., 2018; Ulloa et al., 2019; Vogel et al., 2021). TB effects in the nonsocial condition waned over the course of the experiment (participants became more accurate at replicating intervals), increasing the social-nonsocial difference in block 2. However, contrary to expectations about the shape of the time course, there was no ‘peak’ of binding at any interval (Pfeiffer et al., 2012), nor was there a radically different pattern for social and nonsocial conditions. Instead, percentage binding evened out towards an asymptote, never returning towards more accurate replication at any interval. The effect of socialness did depend on interval length, but subsequent curve fitting showed that this was reflective of a shift in the asymptote and x-intercept, rather than a particular change in the shape of the curve. While there was surprisingly no effect of participants’ beliefs of the social manipulation, this may reflect their unwillingness to admit they had been duped or might indicate their loss of belief towards the end of the long-ish experiment, whereas they actually believed the manipulation for the bulk of the experiment.

## Experiment 2

It is possible that individual differences in baseline time perception could account for some of the between-condition differences in TB observed in Experiment 1. In this experiment we introduced an observation control condition, where participants saw two videos of keypressing hands on the screen and were asked to replicate the interval between them. The same paradigm as in Experiment 1 was used, with some modifications: we used fewer action-response intervals while increasing the highest tested interval to 2,600 ms. The former was so that even with introducing a within-subject control, testing sessions remained around 1 h. The latter was intended to help examine whether there was any decrease in binding at longer intervals.

### Methods

#### Participants

Thirty-four students (23 females; age 17–37 years; mean age = 23.2 years; AQ scores 9–28, mean AQ = 17.4) from the University of Aberdeen participated for renumeration or course credit. Written informed consent was obtained from all participants and the study was approved by the Psychology Ethics Committee, University of Aberdeen (PEC/4504/2020/5). The only a priori inclusion criterion was that participants were naïve to the purpose of the study, i.e., they had not participated in previous similar studies from the research group. Sample size was determined based on initial analyses of Experiment 1, which indicated a larger effect size for Socialness *ƞ2* = .25–.26, and consequently G*power, for *f* = .25 and power = .8, the between-subjects ('Socialness’) effect required a total N = 32.

#### Design

Experiment 2 had a 2 x 2 x 7 mixed design, with a between-subjects factor of ‘Socialness’ (social or nonsocial) and two within-subjects factors of ‘Participant Role’ (action or observation/control) and ‘Interval’ (200–2,600 ms in randomised 400-ms increments; seven intervals total).

#### Materials and Apparatus

**Fig. 4:**
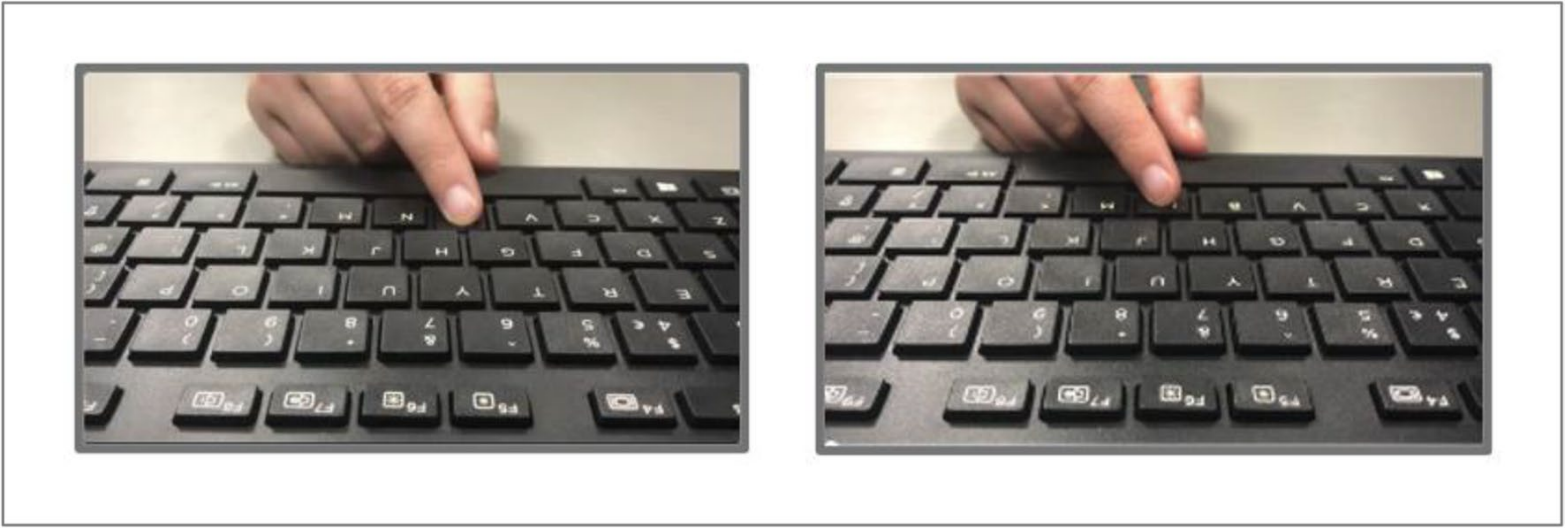
Screenshot of parallel videos viewed by participants in the observation (control) condition of Experiment 2; left video showing hand pressing 'B' key

Materials and apparatus were identical to Experiment 1, except for the following: the control condition showed two videos of a hand and keyboard, each showing a keypress (see Fig. 4). First, the left video would show a hand pressing the ‘B’ key on that keyboard (beginning 500–2,500 ms from trial onset). Then, after a variable delay (200–2,600 ms, in 400-ms increments) after the onset of the first keypress, the right video would show a hand pressing ‘B’ on that keyboard. Both videos would terminate 1,000 ms after the second ‘B’ press. Participants completed the replication task as before, this time for the interval between the two on-screen keypresses.

#### Procedure

Procedure generally followed that for Experiment 1. The main difference was that now participants would not only complete either the assigned social or nonsocial condition, but also a control/“observation” condition (order counterbalanced: before or after the “action” block), in which they did not press any key but viewed two sequential video keypresses (left video press, then right video press; see Fig. 4). This control/“observation” block was not social at any point, but identical in both participant groups. The other difference was that now the AQ was completed between blocks implementing the two ‘role’ conditions (i.e., action and observation); condition order was counterbalanced across participants. It was reasoned that this extended break from replicating intervals would reduce carryover effects from whichever condition was carried out first to the second. There were a few further minor changes: participants now completed a shorter block of practice trials (five trials without interval replication, five trials with interval replication) at the beginning of each ‘role’ condition. The videos used in the nonsocial and control conditions were not of the hand of the researcher present. This allowed greater believability for participants in the ‘live’ interaction social condition as the hand seen on-screen in that condition was the hand of the researcher present, whilst the observation control condition showed a different hand altogether. Finally, with the number of tested intervals reduced, each of the seven intervals were tested six times per block, for two blocks, leading to 84 trials per condition. This meant each participant completed 168 trials total (either social and control conditions or nonsocial and control conditions).

#### Statistical analysis

Outlier detection and exclusion criteria were the same as in Experiment 1. Overall, one participant was excluded (correlation coefficient 2.5 MAD criterion) and 1.9% of trials were excluded (trial-wise 2.5 MAD interval criterion). After all exclusions, there were 15 participants in the social condition (eight females; age 17–37 years; mean age 23.3; AQ scores 10–28, mean AQ = 17.8), and 18 in the nonsocial condition (15 females; age 20–30 years; mean age 22.8 years; AQ scores 9–28, mean AQ = 17.1).

Percentage TB was calculated as in Experiment 1. However, Experiment 2 had a within-subjects control condition. Hence, to adjust for between-participant variability, analyses were carried out on the difference in percentage TB between the interaction conditions (social or nonsocial) and the control condition. Specifically, we subtracted, for each interval, the mean replicated interval in the control condition from each trial in the interaction condition for that interval participant-wise.

The Fixed effects structure of the MLM was the same as in Experiment 1, apart from the following changes. Firstly, as the control and experimental conditions were counterbalanced, a fixed effect of *Position* was added, which accounted for whether the interaction condition came before or after the control for each participant. Secondly, *Belief* was removed as a fixed effect for two reasons: only one participant from the social condition of Experiment 2 reported not believing the ‘social’ interaction, and from the MLM results of Experiment 1, *Belief* did not significantly contribute to the model. The non-believing participant therefore remained in the MLM. The only random effect included in the model was *Participant*, to account for any individual differences in the between-subjects *Socialness* and *Position* factors. The starting point of the MLM was once again the maximal model and the same random effect structure simplification strategy as in Experiment 1 was carried out. *lmer* (Bates et al., 2015) syntax of optimal model reached for Experiment 2 was as follows:

$$Optimal\;Model=lmer\;(percent\;binding\;\left(control\;subtracted\right)\sim1+Interval\;\left(scaled\right)\times Socialness\times Block\times Position+\left(1+Interval\;\left(scaled\right)+Block\left|Subject\right.\right),\;data=Exp2,\;control=lmerControl\;(optimizer=''bobyqa''))$$

### Results

**Fig. 5:**
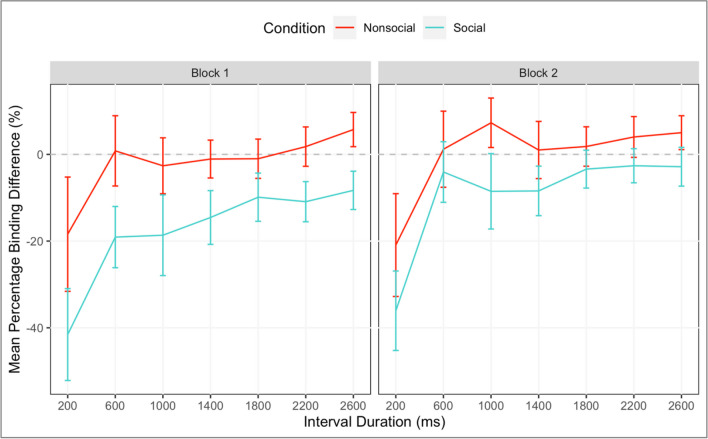
Mean difference scores (experimental – control condition) in percentage temporal binding for each interval within each block of Experiment 2. Green lines show the social condition and red lines show the nonsocial condition. Error bars are 95% confidence intervals

The output of the MLM is shown in Table 2. Figure 5 shows that in line with Experiment 1 results, and crucial to our hypotheses, there was a significant effect of Socialness, with greater binding difference in the social condition (*M = -13.4, 95% CI[-15.2,-11.5]*), compared to the nonsocial condition (*M = -.9, 95% CI[-2.7,1.0]*), *β= 12.73, SE = 5.73, t = 2.22, p = .034.* There was a main effect of Interval on binding difference, *β= 5.97, SE = 1.53, t = 3.90, p < .001,* with the 200-ms interval driving this effect (due to *over-replication* of this interval). There was a main effect of Block on binding difference, *β= 3.59, SE = 1.25, t = 2.88, p = .007,* with responses in block 2 (*M = -4.1, 95% CI[-6.0,-2.3]*) exhibiting less binding difference overall than block 1 (*M = -8.9, 95% CI[-10.8,-7.0])*. All these effects interacted with Position in a significant four-way interaction (*β= -6.91, SE = 3.21, t = -2.15, p = .032)*, where binding difference diminished across blocks, and across the whole experiment binding difference diminished across time from the first to second half, the degree of which depended on interval and condition (see Fig. 5 in OSM for a visualisation of this interaction).

**Table 2:** Results of the mixed linear model to predict temporal binding difference scores (experimental - control conditions) in Experiment 2

**Fixed effect**		***β***		***SE***	***df***		***T***		***p***	
Intercept		-5.82		2.87	28.90		-2.03		.052	
Interval		5.97		1.53	28.79		3.90		<.001	
Socialness		12.73		5.73	28.90		2.22		.034	
Block		3.59		1.25	29.33		2.88		.007	
Position		-10.88		5.73	28.90		-1.90		.068	
Interval*Socialness		-3.99		3.06	28.79		-1.30		.203	
Interval*Block		-.54		.80	2565.41		-.67		.501	
Socialness*Block		-3.62		2.49	29.33		-1.45		.157	
Interval*Position		5.89		3.06	28.79		1.92		.065	
Socialness*Position		2.28		11.47	28.90		.20		.844	
Block*Position		4.20		2.49	29.33		1.69		.102	
Interval*Socialness*Block		.84		1.61	2565.41		.52		.602	
Interval*Socialness*Position		4.20		6.13	28.79		.69		.499	
Interval*Block*Position		-3.31		1.61	2565.41		-2.06		.039	
Socialness*Block*Position		1.28		4.98	29.33		.26		.798	
Interval*Socialness*Block*Position		-6.91		3.21	2565.41		-2.15		.032	
**Random effect**	**Element**		**Variance**			**SD**		**Corr.**		
Participant	Intercept		251.10			15.85				
	Interval(slope)		64.34			8.02		-0.58		
	Block(slope)		28.81			5.37		-0.15		0.21

### Discussion

Experiment 2 confirmed the findings of Experiment 1, demonstrating more TB when participants were told they were interacting with another person in real time as compared to videos across all tested intervals. This experiment introduced a within-participant control condition, which allowed for controlling of individual differences in interval replication. This control condition also showed that TB was reliably observed in both blocks in a social context, whereas TB never significantly differed between nonsocial and control conditions in either block.

Importantly, the main effects of Socialness and Block in Experiment 2 were qualified by a four-way interaction with Position and Interval. In short, the least TB difference between interaction-control was found in (a) the nonsocial condition, (b) at longer intervals, (c) block 2, and (d) when the interaction block was in the second half of the experiment. The fact that binding difference decreased across blocks in the social condition may initially seem like it contradicts the results from Experiment 1, where TB remained constant throughout. However, Experiment 2 used a different measure (within-subject TB difference) from that used in Experiment 1 (TB), so a comparison between the two sets of results must be appropriately cautious. The fact that the main effect of Socialness was observed with both measures, across all tested intervals, despite changes to the experimental design, is testament to the robustness of this effect.

## General discussion

Two experiments used an action-effect interval replication task to investigate if participants’ TB depended on whether they were told they interacted, in real time, with a social partner (social condition) or with a video (i.e., nonsocial condition). The two experiments consistently showed greater TB effects in social, compared to nonsocial, conditions, despite the stimulus remaining the same.

Experiment 1 used a between-subjects design and found that participants in the social condition demonstrated substantially more binding than those in the nonsocial condition, regardless of the action-effect interval or whether they believed the social manipulation. Experiment 2 replicated this difference between social and nonsocial TB, but this time using a within-participant control condition in which participants merely observed two keypresses without own-action initiation. This showed that the social condition represented an increase in TB with respect to the control condition, whereas the nonsocial condition’s binding did not differ from control. Binding effects are implicit: under-replicating intervals goes contrary to task instruction, and in principle, over time participants should learn to accurately replicate those intervals. However, the implicit social binding effect shows no difference between blocks, i.e., it is possible that individual differences in baseline time perception could account for some of the between-subject differences in TB observed in Experiment 1. This may be due to differences in attentional processes between participants in the social and participants in the nonsocial condition, invoked through the change in context (Vogel et al., 2021). This finding that replication accuracy increases (decreasing percentage-binding) with time (block) in the nonsocial condition also argues against fatigue influences (e.g., boredom or rushing to complete the experiment).

The unexpected finding that percentage binding approaches an asymptote, in all conditions, as action-effect intervals increase (i.e., binding measured in milliseconds continues to increase as intervals increase – illustrated by additional analyses presented in OSM in which percentage TB as a function of interval is well fit by a negative exponential curve) supports a theory of TB where the underlying mechanism is the dynamic modulation of the ‘Internal Clock’ (Buonomano, 2007; Gibbon, 1977; Treisman, 1963). The Internal Clock is a neural pacing signal influenced by stimulation and motor activity (Wenke & Haggard, 2009). An individual whose internal clock has a slower oscillatory component would count fewer oscillations over a defined period compared to an individual with a faster oscillation, effectively reducing the perceived time that passed (Fereday & Buehner, 2017). A slowing of this Internal Clock would translate to a consistent *percentage* under-replication of time intervals, as in this experiment.

It is possible that a difference in attentional processes between social and nonsocial conditions is the underlying mechanism that causes the observed modulation of the Internal Clock. The hierarchical model of temporal perception states that ‘subjective duration is known to depend on cognitive load and attentional demand’ with longer perceived durations under greater demands (Pöppel, 1997). This is supported by reduced TB when cognitive loads are greater (Qu et al., 2021). With the current study involving predictable responses, we might speculate that socially predictable responses reduce cognitive load more than similar nonsocial responses. Not only that, but when responses are predictable, it is reasonable to assume that there are more expectations for responses from a person, compared to a computer (Buehner, 2012a, 2012b). Response expectation may, in itself, offer an alternative explanation for the underlying processes behind the time perception modulation found in this study (Hon, 2023; Lelonkiewicz et al., 2020). Hon (2023) acknowledges the role of both attention and expectation in TB effects, but interestingly, for different components: attention underpins action binding, expectation underpins outcome (i.e., effect) binding. These findings were detectable due to the usage of the Libet Clock measure of TB, which requires visual attention to an analogue clockface to mark action and effect positions. If there was a way to develop a non-visual alternative to mark action and effect positions separately, for example an auditory metronome (Silver et al., 2021), it would be of great interest to replicate the current study with such a measurement to isolate potential attention and expectation influences.

The general over-replication of the shortest intervals across conditions may be due to action preparation and/or execution confounds, which can influence the Internal Clock mentioned above. Even when an interval is not initiated by a participant, its replication involves not only an evaluation of the interval, but also assessing the motor production component that signals the start and end of the replicated interval (Gilden et al., 1995). This motor component includes an inherent delay (Gilden et al., 1995), which would explain the ubiquitous over-replication of the shortest intervals. A consistent motor delay would have the greatest impact on the TB of shorter intervals, with the delay effect reducing in influence as intervals increased, which is what our results show. This ubiquitous over-replication aside, the social condition still consistently exhibits relatively shorter interval replications than the nonsocial condition across all intervals.

Our results show notable differences with respect to previous studies. First, whereas we found an overall increase of TB in the social condition, our results differ from the time course of TB obtained by Pfeiffer et al. (2012) for explicit SoA. They found a marked decrease in explicit agency when intervals became longer. We did not observe such a decrease in TB, as the percentage binding in the social condition remained negative, approaching asymptote. Pfeiffer and colleagues (Pfeiffer et al., 2012) used gaze shifts instead of button presses. It is possible that the differences between our findings and theirs could be a consequence of these differences in modality and the measures used (i.e., explicit SoA and implicit TB). As previously mentioned, it is contended within the literature if explicit and implicit measures of SoA correlate or not. It is nonetheless surprising that our results show TB up to 2,100 ms (Experiment 1) and even 2,600 ms (Experiment 2), whereas Pfeiffer et al. (2012) found a consistent linear decline in explicit agency from 400 ms. Future experiments could measure both implicit and explicit SoA to enable direct comparison.

While our results demonstrate greater TB in the social compared to nonsocial condition, they do not reveal its specific underlying causes, nor whether binding is enhanced by the social condition or diminished in the nonsocial condition. Answering these questions would require further investigations and is beyond the scope of this study. This study used a very basic action-reaction interaction without shared goals or intentions other than completing the task. We, therefore, do not know how prosocial consequences entwined with action goals (e.g., establishing joint attention as in Pfeiffer et al., 2012) would impact results. If the underlying mechanism for TB modulation is Sense of Agency, Silver et al.’s (2021) continuum theory of Social Agency suggests that when social effects to our actions are increasingly cooperative, agency effects increase. We could speculate that a more cooperative paradigm would enhance social binding effects, specifically increasing social-nonsocial differences. Conversely, social consequences may also be antisocial (e.g., ostracism). The continuum theory of Social Agency would predict that antisocial action-effects would decrease agency in the social, compared to the nonsocial, condition, particularly for the recipients of such sanctions (Beyer et al., 2017, 2018; Silver et al., 2021).

In our Experiment 2, there was no difference in TB between the nonsocial and observation conditions. This is surprising, in that the nonsocial condition should in principle elicit more TB compared to observation, since it is akin to a standard cause-effect sequence initiated by participant action, which has been widely shown to lead to both explicit and implicit SoA (Moore et al., 2012; Moore & Obhi, 2012; Wen, 2019). It could be that our Experiment 2 showed more than expected binding in the observation condition because the measurement method (i.e., interval replication) still requires voluntary action from the participant, which can, in itself, induce SoA (Haggard, 2017; Haggard & Chambon, 2012). Alternatively, the nature of the nonsocial condition could somehow inhibit TB – for instance due to participants seeing their action as causing the onset of a video in which a hand then pushes a button, rather than feeling they directly caused a hand to press a button. This ambiguity regarding what precisely causes the observed relative differences in the mis-replication of time intervals remains potentially problematic, especially in the light of an absence of an observation versus action difference in the nonsocial condition. As such, investigation of specific social action effects on SoA might require similar experiments such as those presented here using an explicit measure, before we can fully appreciate the mechanisms driving the differences between watching someone react to you, versus a video.

It was unexpected that believing (or not) the social interaction had no effect on binding in Experiment 1. This aspect has previously been overlooked in the field as, generally, participants who do not believe in deceptive interactions are simply excluded (Beyer et al., 2017; Pfeiffer et al., 2012; Stephenson et al., 2018; Vogel et al., 2021). Participants who did not believe the social interaction would be predicted to behave the same as those in the nonsocial condition, since believing the social relevance of on-screen stimuli has been found to be a crucial aspect to socially driven responses (Caruana et al., 2017; Gobel et al., 2018). However, self-report methods cannot be wholly relied upon due to demand characteristics (Nichols & Maner, 2008) and social desirability (Dodaj, 2012). When being told that they had been deceived, it is reasonable to consider that some of the participants who reported not believing the interaction did so out of impression management (Giesen & Rothermund, 2022) whilst genuinely believing. Speculatively, belief may also evolve over the experiment and many “non-believing” participants may have started off, or completed most of, the experiment as believing the cover story, only gradually catching on to the live videos being oddly similar to one another. Importantly, it is unclear if there is a threshold degree of certainty/belief that one is interacting with a conspecific beyond which one experiences *social* TB. Future experiments should continue to consider the role of belief in TB effects, rather than excluding non-believing participants.

In our study the (hand) stimuli could be perceived to be consistently social across all experiments and conditions. Hence, it is possible that there was always an element of social perception, even in the nonsocial conditions (Gobel et al., 2018). Whilst videos of human hands pressing keys can be argued to be social, hands are not as strong a social stimulus as a schematic (Vogel et al., 2021, 2022), an avatar (Pfeiffer et al., 2012) or human (Stephenson et al., 2018) faces that make leading eye movements. Therefore, we do not expect our stimuli to strongly elicit social perception per se. This stimulus difference may explain why the current study’s results diverge from those of Vogel et al. (2021; partially replicated in Vogel et al., 2022). Vogel et al. (2021) did not find an effect of Socialness, that is, whether an interaction was social or not, on TB when the stimulus was a face, whereas our study found a difference in TB between social and nonsocial conditions. Unlike the current study, Vogel et al. (2021) also tested conditions with nonsocial stimulus (i.e., a pattern). Looking at their study as a whole, the nonsocial condition with a social stimulus (face) had far greater TB than the nonsocial condition with the pattern stimulus. This may suggest that a face is such a strong social cue that it overrides the ‘cover story’ (Socialness) manipulation. An avenue of exploration would be to replicate the current experiments with a nonsocial stimulus. This would allow scrutiny of not only social context, but also social appearance, and can examine if these influences interact.

Finally, in both experiments, the other’s response was never uncertain other than temporally (as in many TB paradigms, e.g., Pfister et al., 2014). Hence, the effect of including unpredictable actions on binding cannot be determined from this study (nor was it designed to test this question). However, the cooperation continuum theory of Social Agency would predict diminished binding effects in trials with unpredictable human responses compared to trials with nonsocial responses (Silver et al., 2021). Previous research suggests that expending more cognitive resources to predict the actions of an uncooperative partner interferes with assessing agency, diminishing TB (Beyer et al., 2017, 2018).

## Conclusions

The present study found consistent evidence that (implied) social interactions induce greater TB effects compared to nonsocial interactions, over a wide range of intervals between one’s action and the other’s response. We suggest this difference derives from a modulation of the ‘internal clock’ model of time perception; the clock slows down during social interaction.

## Supplementary Information

Below is the link to the electronic supplementary material.

Supplementary file1 (DOCX 4483 KB)

## Data Availability

Data are deposited on the Open Science Framework at: https://osf.io/usqjy/

## Footnotes

1 Due to COVID-related disruptions, testing took place over many months. The undergraduate researcher testing participants graduated and left the university during this period, therefore they were replaced with a new researcher.

## Code availability

R scripts for analyses deposited on the Open Science Framework at: https://osf.io/usqjy/
