# Supplementary material for: The time course of Temporal Binding in social and nonsocial interactions
Source: Psychon Bull Rev. 2024 Jul 17;32(1):326–41. doi: 10.3758/s13423-024-02540-1 (PMC11836101; doi:10.3758/s13423-024-02540-1)

# Supplementary Materials

## Experiment 1

### Mean interval replication (millisecond) plot

Supplementary Figure 1 shows the mean interval replications for each condition in milliseconds. Replications decrease as interval increases with the social condition consistently exhibiting shorter mean replications than nonsocial.


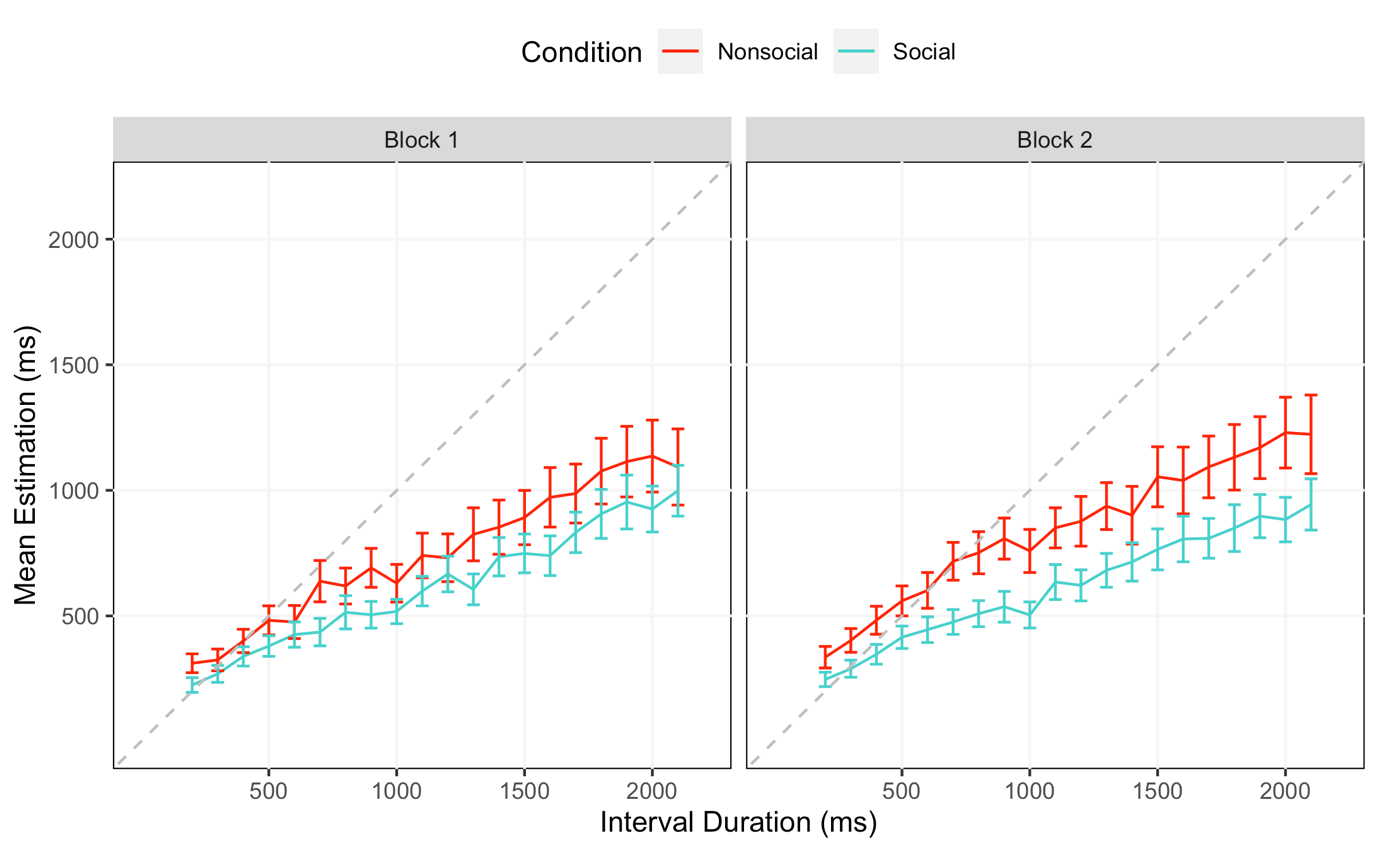


*Supplementary Figure 1*: Mean interval replication (in milliseconds) by interval, block and condition for experiment 1; error bars showing 95% confidence intervals. Dashed line shows where hypothetical accurate replication would lie.

### Age

As Experiment 1 included between-subjects variables, it was important to rule out the possibility of demographic differences between conditions contributing to any TB effects. For age, the nonsocial condition had a much higher mean (*29.1, SD = 11.6*) than both social conditions (social_yes, *M = 22.1, SD = 2.8*; social_no, *M = 22.2, SD = 2.7*), as shown in Supplementary Table 1.

*Supplementary Table 1: Mean age for each Cond_Bel condition with standard deviations in parentheses.*

| **Cond_Bel condition** | **Mean Age (SD)** |
| --- | --- |
| nonsocial | 29.1 (11.6) |
| social_yes | 22.1 (2.8) |
| social_no | 22.2 (2.7) |

To rule out any positive association between age and the greater TB effects found in the nonsocial condition of the MLM for experiment 1, a one-tailed correlation between age and mean TB (across all intervals) was carried out. As data was not normally distributed Spearman’s correlation was used. Results showed there was a weak, non-significant positive association between age and mean TB *r_s_*(29) = .28, *p* = .147 (Supplementary Figure 2).


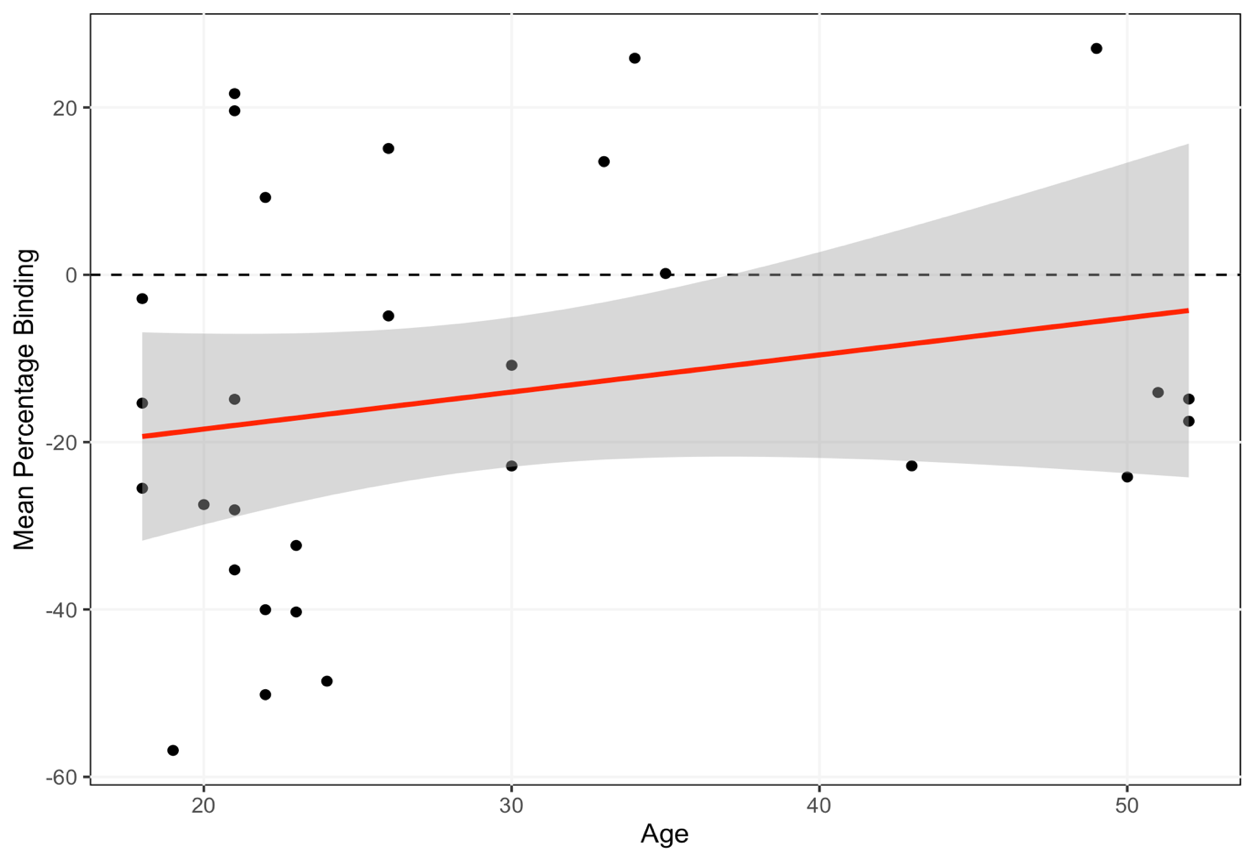


*Supplementary Figure 2*: Scatterplot showing a non-significant weak positive association between age and mean percentage TB for the nonsocial condition of the MLM for Experiment 1. Line of best fit in red, 95% confidence interval represented by the shaded area.

### Curve fitting to examine the Socialness/Belief*Interval interaction

To examine the time course of binding differences between social and nonsocial conditions (Socialness/Belief*Interval interaction), we subjected the data from Experiment 1 to curve-fitting; exponential curves were fit to percent binding as a function of Interval for each participant. The parameters of these curve fits were then compared across conditions using analyses of variance (ANOVAs). The exponential function (Rashal & Yeshurun, 2014; Scolari et al., 2007; Soo et al., 2018) used to fit the data was:

$y\left( x \right)= \propto({1-e}^{\left( -s\left( x-t \right) \right)})$ (2)

where $y$ is the interval-replication percentage binding, $\propto$ is the asymptote, $s$ is the scaling factor, $x$ is the interval and $t$ is the x-intercept of the curve (see Supplementary Figure 3 for a visualisation of the model). Critical interval ($x$_c_) was defined as the interval at which percentage binding reached 90% of the asymptote, and was calculated using the following equation (3) for each Socialness/Belief condition:
$x_{c}=t-log(0.1)/s$ (3)


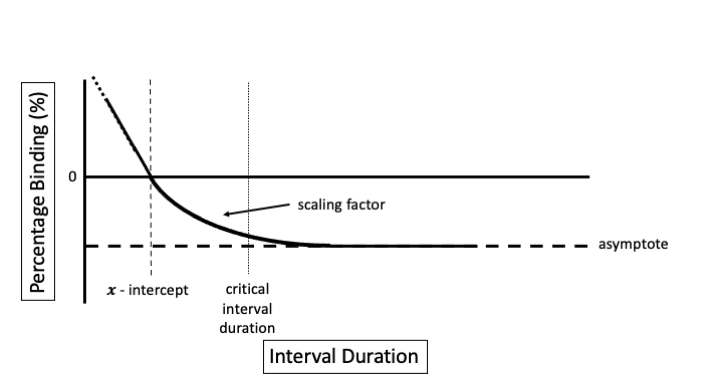


*Supplementary Figure 3*: Illustration of a negative exponential curve along with the geometric interpretation of the parameters are shown.

Any curves with poor fits (r^2^ ≤ .3) were excluded from the subsequent analysis; in total 8 participant curves (3 nonsocial, 2 social_no, 3 social_yes) were excluded. Exponential curve fits to the mean data (averaged over participants) for each condition are shown in Supplementary Figure 4, panel A. Analyses of variance were then carried out across the between-subject Socialness/Belief conditions on the asymptotes, x-intercepts and critical intervals.

A one-way between-subjects ANOVA was carried out on the asymptotes of the three Socialness/Belief conditions. There was a significant effect of condition, *F(2, 53) = 5.43, p = .007, η^2^_p_ = .17*. Bonferroni corrected comparisons revealed that the difference between nonsocial (*M = -40.0, 95% CI [-49.4, -30.5]*) and social non-believers (Social No in Supplementary Figure 4; *M = -60.8, 95% CI [-71.7, -50.0]*) was significant, *t(53) = 3.00*, *p = .012, d = 1.0*. No other comparisons were significant: the difference between nonsocial and social believers (Social Yes in Supplementary Figure 4; *M = -54.4, 95% CI [-62.5, -46.3]*) was p = 0.06, the difference between social non-believers and social believers was p = 1. Similarly, a one-way between-subjects ANOVA on the x-intercepts of the Socialness/Belief conditions indicated a significant effect of condition, *F(2, 53) = 7.20, p = .002, η^2^_p_ = .21.* Bonferroni corrected planned comparisons revealed that the difference between nonsocial (*M = 489.7, 95% CI [403.5, 576.0]*) and social believers (Social Yes in Supplementary Figure 4; *M = 272.0, 95% CI [200.3, 343.7]*) was significant, *t(53) = 3.64, p = .002, d = 1.2*. No other comparisons were significant: the difference between nonsocial and social non-believers (Social No in Supplementary Figure 4*; M = 326.3, 95% CI [ 177.4, 475.2])* was p = 0.07, the difference between social non-believers and social believers was p ≈ 1. Finally, a one-way between-subjects ANOVA on the critical intervals of the Socialness/Belief conditions did not reveal any significant differences, *F(2, 53) = 0.51, p = .604, η^2^_p_  = .02*. All three parameters are plotted in Supplementary Figure 4.


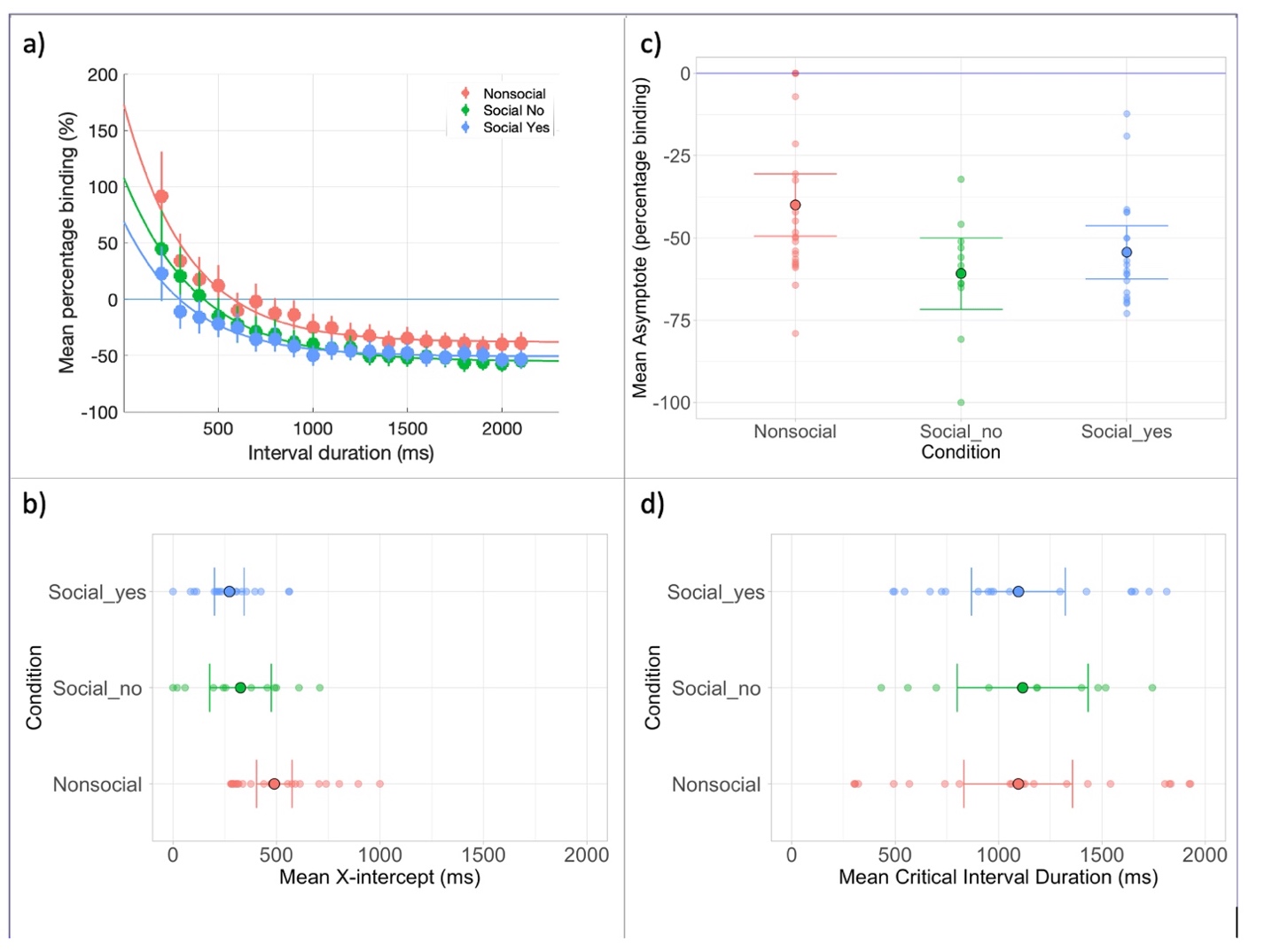


*Supplementary Figure 4*: a) exponential curves fitted average percentage TB as a function of interval for each Socialness/Belief condition; b) Mean x-intercept in milliseconds by condition; c) Mean Asymptote in percentage binding by condition; d) Mean critical Interval in milliseconds by condition (error bars = 95% confidence intervals throughout). Opaque points plot conditions means throughout; semi-transparent points plot individual participant values across panels b) – d).

## Experiment 2

### Mean TB difference interaction plot

Supplementary Figure 5 shows the significant 4-way interaction from Experiment 2, where participants exhibit more binding both at the beginning of the experiment and at the beginning of each condition (block 1), depending on both the condition position and interval.

*Supplementary Figure 5*: Mean percentage TB difference by interval, block, condition and position for experiment 2; error bars showing 95% confidence intervals.


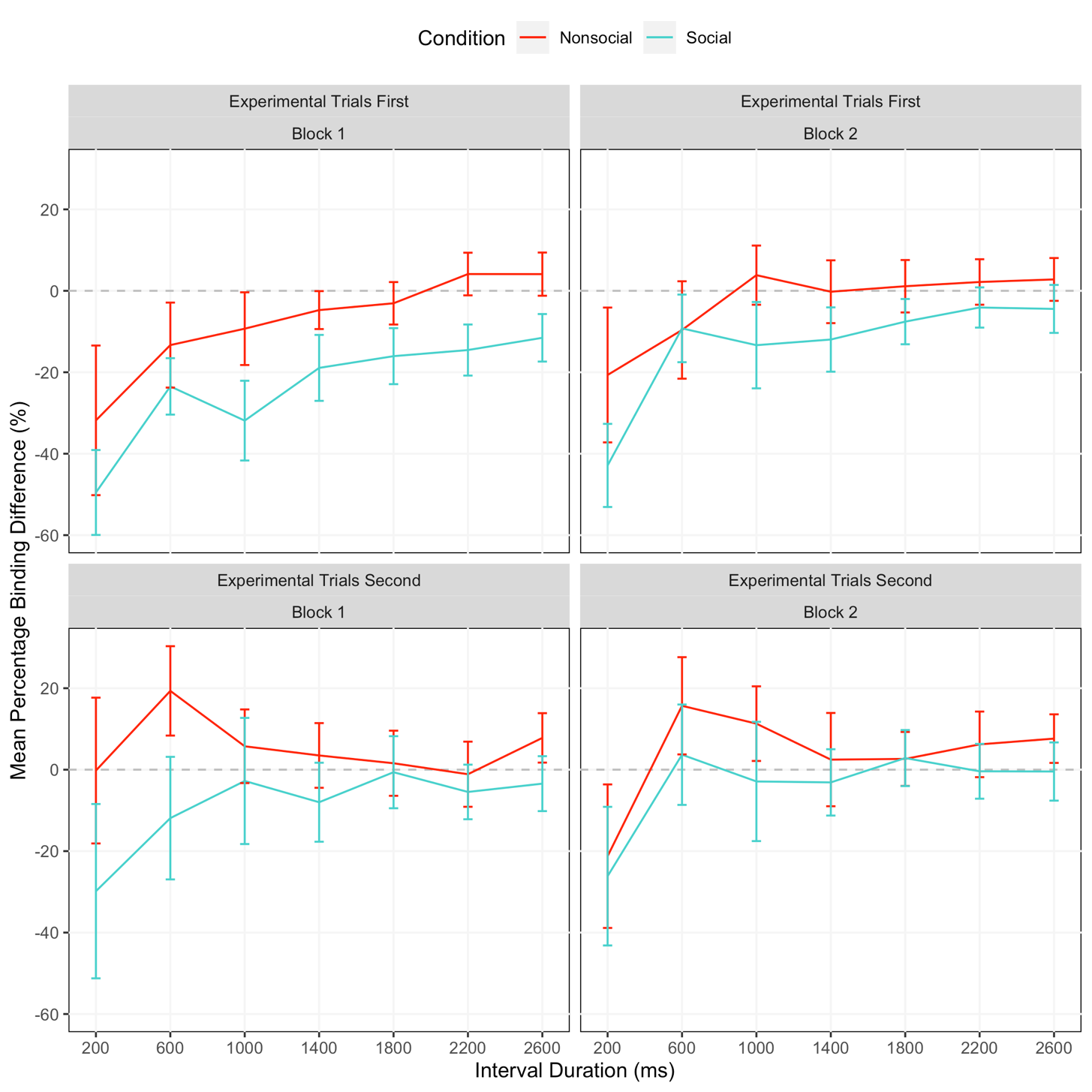


### Mean replication (milliseconds) plot

Supplementary Figure 6 shows the mean interval replications for experimental and control trials by interval, condition, block and whether the experimental trials came first or second.


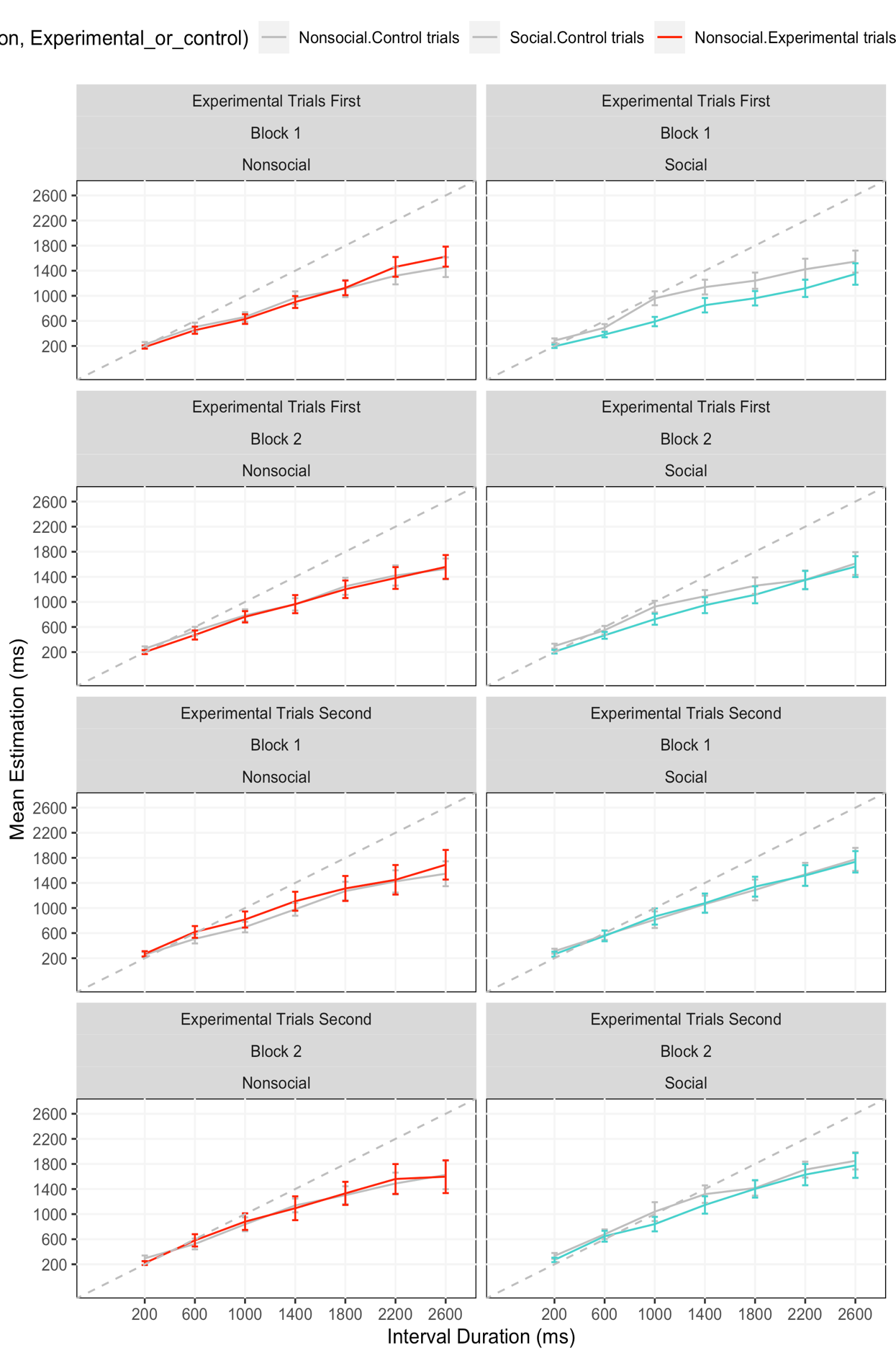


*Supplementary Figure 6*: Mean interval replication (in milliseconds) by interval, block, condition, position, and trial type (control or experimental) for experiment 2; error bars showing 95% confidence intervals. Control means are depicted in grey lines, with social means in green and nonsocial means in red. Dashed line shows where hypothetical accurate replication would lie.


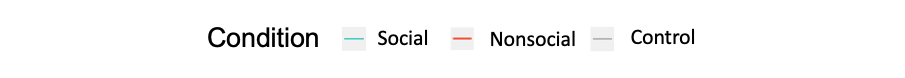


### Mean TB plot

Supplementary Figure 7 shows the TB for each condition (i.e., without transforming into difference scores), by interval, block and whether the experimental trials came first or second, where, as in Experiment 1, TB increases as interval increases, approaching negative asymptote.


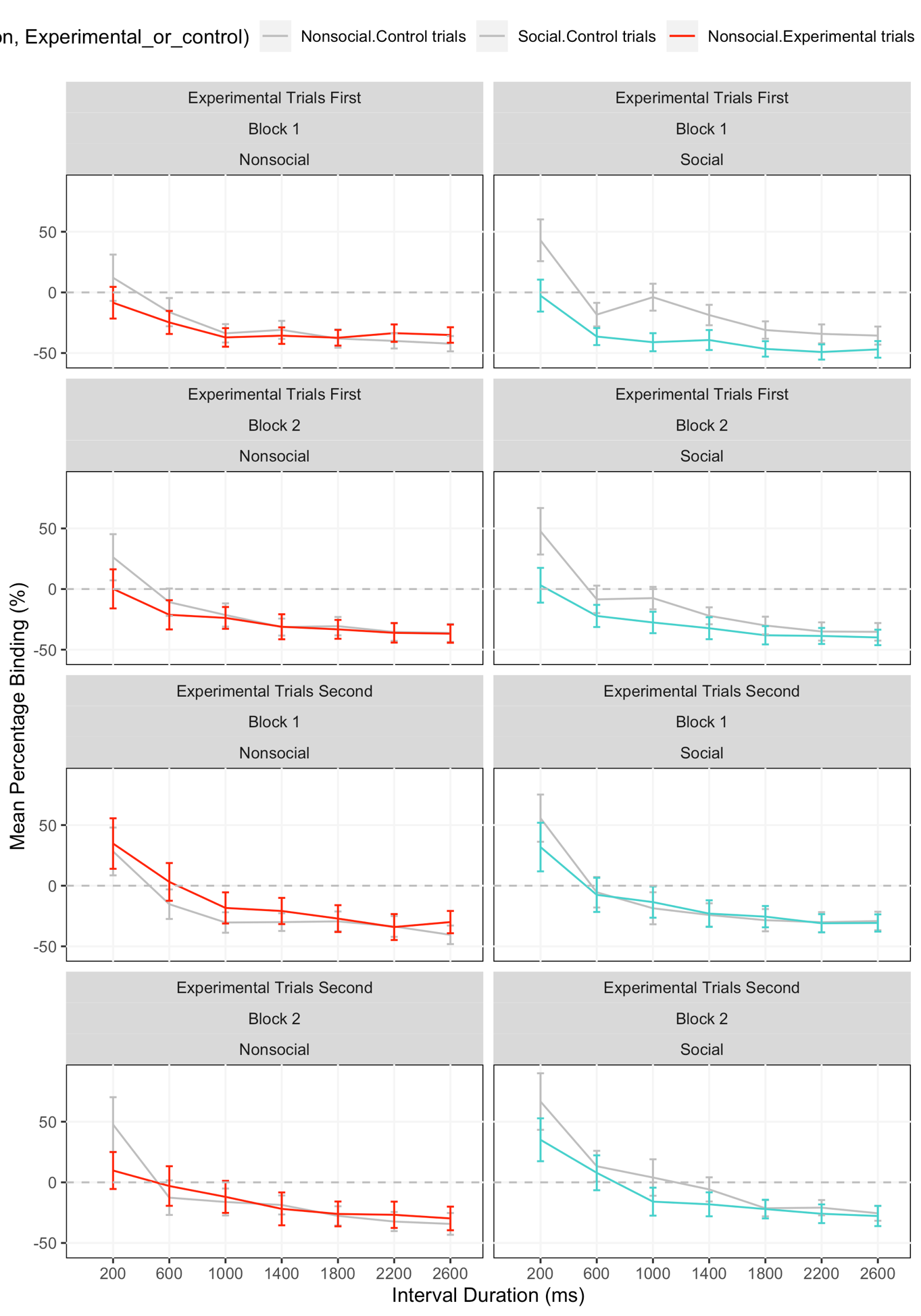


*Supplementary Figure 7*: Mean percentage TB by interval, block, condition, and position for experiment 2; error bars showing 95% confidence intervals. Control means are depicted in grey lines, with social means in green and nonsocial means in red. Dashed line shows where hypothetical accurate replication would lie.


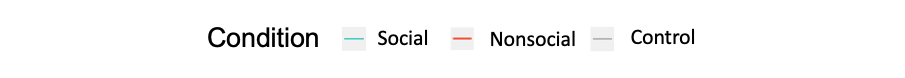

Supplement: Supplementary file 1 — Supplementary file1 (DOCX 4483 KB) [file 13423_2024_2540_MOESM1_ESM.docx]
